# Assessment of Midwife Knowledge, Practice, and Associated Factors towards Active Management of the Third Stage of Labor at Governmental Health Institutions in Tigray Region, Northern Ethiopia, 2018

**DOI:** 10.1155/2020/8547040

**Published:** 2020-11-03

**Authors:** Getu Engida Wake, Girma Wogie

**Affiliations:** ^1^Department of Midwifery, Debre Berhan University, Debre Berhan, Ethiopia; ^2^Department of Midwifery, Wollo University, Wollo, Ethiopia

## Abstract

**Introduction:**

Globally, postpartum hemorrhage is the most common cause of maternal mortality and morbidity, and it accounts for more than 25% of all maternal deaths. The majority of death due to postpartum hemorrhage is caused by uterine atony. Routine and correct usage of active management of the third stage of labor decreases the occurrence of postpartum hemorrhage by 60% when compared to expectant management of the third stage of labor. The purpose of this study was to assess midwife knowledge, practice, and associated factors towards active management of the third stage of labor at governmental health institutions in the Tigray region, 2018.

**Results:**

These study results showed that from the total study participants (*N* = 278), 170 (61.2%) were good in knowledge and 121 (43.5%) were good in practice towards active management of the third stage of labor. Training related to active management of the third stage of labor (AOR = 2.119, 95%CI = 1.141, 3.3937) and practice level of midwives (AOR = 8.089, 95%CI = 4.103, 15.950) became significantly associated with the knowledge level. The educational level of midwives (AOR = 3.811, 95%CI = 2.015, 7.210), training related to active management of the third stage of labor (AOR = 2.591, 95%CI = 1.424, 4.714), and knowledge level of midwives towards active management of the third stage of labor (AOR = 7.324, 95%CI = 3.739, 14.393) were significantly associated with the practice level. This study showed that training related to active management of the third stage of labor was significantly associated with the knowledge and practice level of midwives. The educational level and knowledge level of midwives were significantly associated with the practice level of midwives towards active management of the third stage of labor. Therefore, midwives should update their academic level and knowledge. Health institutions in collaboration with the Tigray Regional Health Bureau should arrange training for all midwives to bring change.

## 1. Introduction

Labor is defined as consecutive events that take place in the genital organs to expel products of conception out of the womb through the vagina into the outer world. The process of labor involves four stages. The first stage starts from the onset of true labor and ends with full dilatation of the cervix. The second stage is from the full dilatation of the cervix to the expulsion of the fetus. The third stage is the stage of labor that is demarked by the delivery of the fetus and expulsion of the placenta and membrane, which lasts from five to fifteen minutes and the most hazardous stage of labor due to the risk of profuse bleeding. And the fourth stage is the stage of labor that extends to one hour after delivery of the placenta [1].

The main complication associated with the third stage of labor is postpartum hemorrhage (PPH) which is generally described as blood loss greater than or equal to 500 ml within 24 hours after birth, but in severe condition, blood loss is greater than or equal to 1000 ml within 24 hours [2].

Developing countries account for a higher percentage of global maternal mortality estimate of 2015, and they account for 99% (302,000) of the estimated global maternal deaths in 2015, with sub-Saharan Africa alone accounting for roughly 66% (201,000). The overall MMR in developing regions is 239 per 100,000 live births which is much higher than that of developed regions, where it is just 12 per 100,000 live births, and sub-Saharan Africa has a very high maternal mortality rate (MMR) of 546 per 100,000 live births [3, 4].

Every year, more than a half-million women die from complications of pregnancy and childbirth, and most of this death occur in developing countries due to many factors mostly such as inability to access quality emergency obstetrical services, lack of transportation, lack of awareness, lack of trained health professionals, and other factors [5, 6].

A large number of maternal death related to pregnancy and childbirth are due to postpartum hemorrhage. According to the World Health Organization (WHO), it accounts for worldwide annual 127,000 maternal deaths. Even if PPH is a preventable and manageable problem in a developed country, it accounts for 60% of all maternal deaths in developing countries [7].

Similar to developing countries, trends of maternal mortality in Ethiopia are also very high. The present estimates of the pregnancy-related mortality ratio are approximately 412 per 100,000 live births. Although there is a decrement of the maternal mortality rate from 2000 to 2015 which was from 871 to 412, still we are within the range of the high maternal mortality rate [8].

Even though it is not easy to find the correct number of maternal death secondary to postpartum hemorrhage (PPH), it is estimated that around 25-30% maternal death was due to PPH in Ethiopia [9].

Maternal death due to postpartum hemorrhage occurs in health facilities of low-income countries where there are no adequate obstetric care providers or where they lack the necessary knowledge, skill, and supplies to prevent and manage the problem [10].

The majority of death due to postpartum hemorrhage (PPH) is caused by uterine atony (a condition when the uterine muscle failed to contract and legate uterine blood vessels after delivery of the placenta). Most of the time, maternal mortality and morbidity due to postpartum hemorrhage (PPH) take place within the first 24 hrs after delivery within the time frame of primary PPH. Around 88% of maternal death due to postpartum hemorrhage takes place within the first 4 hrs of delivery which indicates the severity of this period because of complications of the third stage of labor [11–13].

The two known protocols used for management of the third stage of labor are the active management of the third stage of labor and expectant (physiological) management of the third stage of labor. Active management of the third stage of labor involves the provision of prophylactic uterotonics, controlled cord traction for delivery of the placenta, and immediate uterine fundus massage unlike that of expectant management of the third stage of labor [14].

Active management of the third stage of labor is recognized globally and supported for more than decades as a management package for reduction of maternal mortality and morbidity secondary to postpartum hemorrhage in many countries, and it is incomparable to that of expectant management of the third stage of labor in which the risk of postpartum hemorrhage (PPH) is more than 60% lower when we compare it with expectant management of the third stage of labor. It is the only option which is easy, cost-effective, and applicable management package for the third stage of labor to prevent postpartum hemorrhage and its complications, and this management package is aimed at hastening delivery of the placenta by increasing the frequency and strength of uterine contraction, preventing the occurrence of uterine atony and other causes of postpartum hemorrhage, and minimizing the numbers of maternal death [4, 15].

Even if active management of the third stage of labor (AMTSL) plays a great role in the prevention and management of postpartum hemorrhage, there is a gap of knowledge and practice of midwives towards active management of the third stage of labor [16, 17]. However, little has been known about midwife knowledge, practice, and associated factors towards active management of the third stage of labor in our country, and no study is conducted in the current study area, so this study was aimed at finding out midwife knowledge, practice, and associated factors towards active management of the third stage of labor in the Tigray region.

## 2. Methods and Materials

### 2.1. Study Design and Period

Institutional-based cross-sectional study design was conducted from November 15/2017 to January 12/2018 to assess midwives' knowledge, practice, and associated factors towards active management of the third stage of labor.

### 2.2. Study Area and Population

The study was conducted at all governmental health institutions found in two zones of the Tigray region. The Tigray region is the northernmost of the nine regions of Ethiopia. It is also known as Region 1 according to the federal constitution. The state's capital and largest city is Mekelle. Tigray is bordered by Eritrea to the north, Sudan to the west, Afar region to the east, and the Amhara region to the south and southwest [18]. Based on the projection made from the Ethiopian census of 2007, the region had a total population of 4,806,843 of whom 2,441,158 (50.8%) were female in 2015. The region is administratively divided into 7 zones including one special zone (Mekelle). The Tigray regional state has a total of 24 hospitals and 254 health centers, and the total number of midwives in the region is 918. The study was conducted in two zones of the Tigray region, the central zone and Mekelle specialized zone which contains 73 governmental health institutions (62 health centers and 11 hospitals), and the total number of midwives in the two zones was 304 [19]. So, all midwives who were working in the delivery room of selected governmental health institutions (62 health centers and 11 hospitals) in the Tigray region during data collection were included.

### 2.3. Sample Size and Sampling Procedure

The sample size was determined using a single population proportion formula at 95% confidence interval with the assumption of the prevalence of AMTSL knowledge in Ethiopia 37.7% [20]. With *α* = 0.05, marginal error *d* = 0.05. After using the correction formula, the final sample size became 285 midwives. Seven zones of the Tigray region were clustered, and two of them (30%) were taken by simple random sampling, and all midwives (285) working in the governmental health institutions (11 hospitals and 62 health centers) that are found in the two selected zones of the Tigray region who fulfilled the inclusion criteria were included in the study.

### 2.4. Data Collection Tool and Procedure

Data was collected by face-to-face interview questionnaires and observation by using a semistructured questionnaire and observational checklist. Semistructured questionnaires were adapted and adopted from different literature, while the observational checklist was adopted from ICM and FIGO guidelines. The face-to-face interview using a questionnaire was used to assess study participant sociodemographic information and knowledge, and an observational checklist was used to assess midwife practice. Data was collected by 20 degree midwives who had experience in data collection and 3 supervisors.

### 2.5. Data Quality Assurance

Questionnaires and checklist were prepared in the English language by the principal investigator and reviewed by the advisors. These questionnaires and checklist were pretested on 10% of the calculated sample size outside the selected study area (Machew Hospital, Adigudom Primary Hospital, and Hewane Health Center), and one-day training was given by the principal investigator for data collectors and supervisors concerning the research objective, data collection tools, procedures, and how to fill the questionnaire and checklist properly. Moreover, data quality was assured by designing a data collection instrument, and 10% of the collected data was checked by the supervisor daily for completeness, and finally, the principal investigator monitored the overall quality of data collection.

### 2.6. Data Entry and Analysis Procedure

The collected data was cleaned, coded, and entered into Epi Info version 3.5.1 and transported to SPSS (Statistical Package for the Social Sciences) version 20 for analysis. Descriptive statistical analysis was used to compute frequency, percentage, and others such as measures of central tendency. Binary logistic regression analysis was used to identify the association between dependent and independent variables. Variables with a significant association in the bivariate analysis were entered into multivariate analysis to determine the knowledge and practice of midwives towards AMTSL, and variables with *p* value less than 0.2 and 0.05 were considered statistically significant for bivariate and multivariate regression, respectively. The overall results were presented in texts, tables, and figures.

### 2.7. Independent Variables

These are sociodemographic characteristics such as religion, ethnicity, age, sex, and others such as year of experience, place of work, level of education, in-service training, access to reading material, and availability of uterotonic drugs.

### 2.8. Dependent Variables

The dependent variables are knowledge and practice.

### 2.9. Operational Definitions

Knowledge refers to the level of awareness and understanding of midwives regarding active management of the third stage of labor. Good in knowledge is defined as those who knew 5 and above from seven questions prepared to assess the knowledge level of midwives towards AMTSL. Poor in knowledge is defined as those who answered less than five questions from seven questions prepared to assess knowledge of midwives on AMTSL.

Practice refers to the ability of midwives to carry out the management of the third stage of labor. Good in practice is defined as those who performed13 steps correctly in the proper sequence from 15 steps prepared to assess midwives' skill. Poor in practice is defined as those who performed less than 13 steps correctly in the proper sequence from 15 steps prepared to assess midwife skill.

## 3. Results

### 3.1. Sociodemographic Characteristics of Midwives

From a total of 285 study subjects, 278 midwives participated in the study with a response rate of 97.5%. The majority of the 119 (42.8%) were between 31 and 40 years old. The mean age of study participants was 35 years ± 7.28 (SD). More than half of the midwives (215, 77.3%) were female, and almost all of the study participants were Orthodox religion followers and Tigray in ethnicity. Regarding their marital status, 188 (67.6%) were married and 156 (56.1%) were diploma midwives. More than half of them (181, 65.1%) were from hospitals and 155 (55.8%) had no training related to AMTSL. From the total study participants, 111 (39.9%) had 5-9 years of experience years in attending labor and 146 (52.5%) reported that they did not have reading material related to AMTSL in their health institutions. All of them (278, 100%) had uterotonics in their health facilities (Tables 1 and 2).

### 3.2. Knowledge of Midwives on Active Management of the Third Stage of Labor

**Figure 1:**
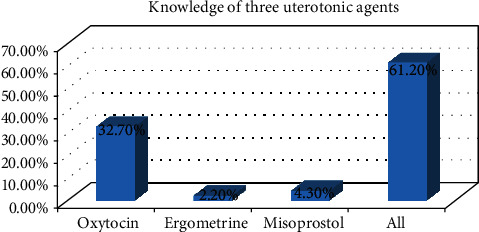
Knowledge of midwives towards three uterotonic agents in governmental health institutions in the Tigray region, Ethiopia, 2018.

The study found that from the total midwives included in the study (*N* = 278), more than half of them (170, 61.2%) were good in knowledge towards AMTSL and 108 (38.8%) were poor in the knowledge of AMTSL. The majority of them (230, 82.7%) knew all the essential components of AMTSL. Around 246 (88.5%) correctly answered the recommended dose of oxytocin for the management of AMSTL as 10 IU. The majority of midwives (246, 88.5%) knew the time of administration of oxytocin for management of AMTSL which is within one minute of delivery of the baby. More than half of them (179, 64.4%) knew that the frequency of uterine massage after delivery of the baby is every 15 minutes for the 1^st^ 2 hrs (Table 3 and Figure 1).

### 3.3. Practices of Midwives on Active Management of the Third Stage of Labor

**Figure 2:**
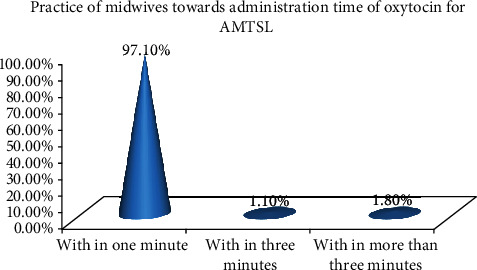
Practice of midwives towards administration time of oxytocin for management of AMTSL in governmental health institutions in the Tigray region, Ethiopia, 2018.

In this study, the overall good practice and poor practice of midwives towards active management of the third stage of labor were 121 (43.5%) and 157 (56.5), respectively. All of them (278, 100%) were observed while they administer 10 IU of oxytocin in the IM route of administration. Almost all of them (270, 97.1%) gave oxytocin within one minute of the delivery of the baby. The majority of them (199, 71.6%) correctly applied CCT as per protocol. Regarding immediate uterine massage and extraction of the membrane gently with lateral movement, the majority, 206 (74.1%) and 199 (71.6%), of midwives, respectively, were observed while they did the procedure correctly (Table 4 and Figure 2).

### 3.4. Factors Associated with Knowledge of Midwives towards AMTSL

On multiple logistic regressions, training related to AMTSL and practice level of midwives became significantly associated with the knowledge level. Accordingly, those midwives who had training related to AMTSL were 2 times more likely to be knowledgeable when compared with their counterparts (AOR = 2.119, 95%CI = 1.141‐3.3937). Those midwives who were good in practice were 8 times more likely to be knowledgeable than those who were poor in practice towards AMTSL (AOR = 8.089, 95%CI = 4.103‐15.950) (Table 5).

### 3.5. Factors Associated with the Practice of Midwives towards AMTSL

On multiple logistic regression analysis, three variables (educational level of midwives, training related to AMTSL, and knowledge level of midwives towards AMTSL) were significantly associated with the practice level of midwives. Degree midwives were 4 times more likely to be good in practice than diploma holders (AOR = 3.811, 95%CI = 2.015‐7.210). Those midwives who took training related to AMTSL were 3 times more likely to be good in practice than their counterparts (AOR = 2.591, 95%CI = 1.424‐4.714). Moreover, those midwives who were good in knowledge were 7 times more likely to be good in practice than those who were poor in knowledge (AOR = 7.324, 95%CI = 3.739‐14.393) (Table 6).

## 4. Discussion

The main aim of this study was to assess midwife knowledge, practice, and associated factors towards active management of the third stage of labor at governmental health institutions in the Tigray region, 2018. In this study, the overall knowledge and practice of midwives towards active management of the third stage of labor were 61.2% and 43.5%, respectively. This finding is in line with the study conducted in Nigeria and Ethiopia, Addis Ababa, which showed that the overall knowledge and practice of midwives towards AMTSL were 66.7%, 41.7% and 51.5%, 47%, respectively [21, 22].

This might be due to the similarity in sociodemographic status of study participants in which midwives from both health center and hospitals were included in the study but higher than the study conducted in Ethiopia, Hawassa, and study conducted in Sudan which showed that 33.3%, 16.7% and 15.6%, 26.7% of study participants were good in knowledge and good in practice, respectively [16, 17]. This difference might be due to the difference in the educational level of study participants conducted among midwives where there were a large number of diploma holders.

Again, the finding of this study was much higher than the result of the study conducted in Tanzania which showed that the overall knowledge and practice of active management of the third stage of labor were only 10% for both [23]. This significant difference might be due to the educational level of study participants in Tanzania; from 87 midwives involved in the study, only one midwife was a degree holder.

This study result regarding overall knowledge of midwives towards AMTSL was much lower than the study result conducted in Southwest Nigeria which showed that 90.6% of the respondents had good knowledge in AMTSL [24]. The study was conducted only in public tertiary hospitals, and this might be what brought the difference.

This study found that knowledge of study participants concerning the recommended time of oxytocin administration which is within one minute of delivery of the baby as per guideline of FIGO/ICM was 88.5%. This finding is higher than the result of the study conducted in Sudan which showed that only 48% knew the variable [25]. This difference might be due to the absence of training and educational level of study participants in the study area.

Regarding knowledge of three essential components of AMTSL in this study, 82.7% of the study participants knew all of the essential components. This result is much higher than the result of the study conducted in Tanzania which showed that only 9% of the study participants were knowledgeable on three essential components of AMTSL [25]. This difference might be due to the time difference between these two studies and the difference in the national guideline.

Concerning the recommended dose and route of oxytocin administration knowledge, this study found that 88.5% and 88.1% of midwives were knowledgeable, respectively. This finding is similar to the study conducted in Hawassa City, Ethiopia, which indicated that 86.1% and 81.9% of the study participants were knowledgeable [16]. This study showed that those midwives who took training related to AMTSL were 2 times more likely to be knowledgeable compared to their counterparts and those midwives who were good in practice were 8 times more likely to be knowledgeable when compared to those midwives who were in a poor practice level. This result is in line with the study conducted in Addis Ababa which showed a significant association between midwives' practice level and knowledge [26]. This similarity might be due to the fact that those who had good skills update themselves daily and reason out why and how they perform every step in their skill.

This study found that around 64.7% of the study participants palpated the maternal abdomen to rule out the presence of another fetus before administration of the uterotonic drug. This is in line with the result of the study conducted in Ethiopia which was 65% [16]. This similarity might be due to the characteristics of the study area, which include both hospital and health center midwives.

But it is lower than the result of the study conducted in Ethiopia (82.4%) and another study conducted in Ethiopia (82.3%) of study participants who had palpated the maternal abdomen before the administration of uterotonic drugs [22, 26]. Both of the studies conducted in the capital city of Ethiopia, Addis Ababa, brought the difference.

This study showed that completeness of the placenta and membrane and assessing maternal genitalia for tear and trauma were assessed in 67.3% and 74.5%, respectively. This result is lower than the result of the study conducted in Nepal which showed 98.1% and 97% of the study participants who were observed while they did, respectively [27]. This difference might be due to the difference in the study area, which was conducted only in the hospital which was a training center.

According to this study result, around 74.1% of the study participants were observed while they immediately massage the uterus after delivery of the placenta. This finding is in line with the study conducted in Southwest Nigeria (73.3%) and Sudan [17, 21]. This might be due to the similarity of the sociodemographic status of the study participants in which midwives working in hospitals and health centers were included especially for the first research.

The majority of these study participants (75.5%) were observed while they ensured that the uterus did not relax after stopping the uterine massage. This study result is in line with the study conducted in Ethiopia which showed that 74.3% of midwives have performed the procedure [22].

According to this study result, the overall practice of AMTSL is 43.5%. This result is in line with the study conducted in Netherlands (48%) and Nigeria (42%), respectively [28, 29], but much higher than the result of the study conducted in Tanzania and study conducted in some rare developing countries in which only 7% and 5-32% were good in practice towards AMTSL, respectively [25, 30]. This difference might be due to the time difference at which the study was conducted and the national guideline of Tanzania which supports the administration of 0.5 mg IM ergometrine or 5 IU of oxytocin rather than 10 IU of oxytocin for active management of the third stage of labor.

The educational level of midwives, training related to AMTSL, and knowledge level of midwives were significantly associated with the midwives' practice level. Those degree midwives were 4 times more likely to be good in practice towards AMTSL than diploma holders. Those midwives who took training related to AMTSL were 3 times more likely to practice active management of the third stage of labor than their counterparts. Those midwives who were in good knowledge levels were 7 times more likely to practice AMTSL when compared to those with a poor knowledge level. This result is in line with the result of the study conducted in Ethiopia which showed the association of the educational level, training, and knowledge level of midwives towards active management of the third stage of labor [26].

## 5. Conclusion

The overall knowledge and practice of midwives towards active management of the third stage of labor in this study were in line with the study conducted in Nigeria and the study conducted in Addis Ababa, Ethiopia. Training related to active management of the third stage of labor and practice level of midwives towards active management of the third stage of labor were significantly associated with the knowledge level of midwives. The educational level, training related to active management of the third stage of labor, and knowledge level of midwives were significantly associated with the practice level of midwives towards active management of the third stage of labor. Midwives should update their academic level and knowledge and improve their skills to provide fruitful service towards AMTSL and save mothers' lives. Health institutions should arrange training for all midwives, and the regional government should upgrade the midwives' educational level.

## Figures and Tables

**Table 1:** Sociodemographic characteristics of midwives in governmental health institutions in the Tigray region, Ethiopia, 2018.

Variables		Frequency (*n* = 278)	Percentage (%)
Age	20-30	107	38.5
	31-40	119	42.8
	41-50	45	16.2
	Greater than 50	7	2.5

Sex	Female	215	77.3
	Male	63	22.7

Religion	Orthodox	277	99.6
	Muslim	1	0.4

Ethnicity	Tigray	278	100

Marital status	Single	69	24.8
	Married	188	67.6
	Divorced	18	6.5
	Widowed	3	1.2

Educational level	Diploma midwife	156	56.1
	Degree midwife	122	43.9

Workplace	Health center	97	34.9
	Hospital	181	65.1

Years of experience in attending labor	6 months-4 years	102	36.7
	5 years-9 years	111	39.9
	Greater than 9 years	65	23.4

**Table 2:** Training related to AMTSL, availability of reading materials, and availability of uterotonic drugs in governmental health institutions in the Tigray region, Ethiopia, 2018.

Variables		Frequency (*n* = 278)	Percentage (%)
Training related to AMTSL	No	155	55.8
	Yes	123	44.2

Availability of reading material in health institutions	No	146	52.5
	Yes	132	47.5

Availability of uterotonic drugs	No	0	0
	Yes	278	100

**Table 3:** Knowledge of midwives towards active management of the third stage of labor in governmental health institutions in the Tigray region, Ethiopia, 2018.

Variables	Response options	Frequency (*n* = 278)	Percentage (%)
Essential components	(i) Administer uterotonic drugs	34	12.2
	(ii) Apply CCT	6	2.2
	(iii) Uterine massage	8	2.9
	(iv) All	230	82.7

Role immediately after delivery of the 1^st^ baby	(i) Administer uterotonic drugs	91	32.7
	(ii) Check the presence of another baby	170	61.2
	(iii) Uterine massage	17	6.1

Recommended dose of oxytocin for active management of third stage of labor	(i) 0.5 mg	14	5
	(ii) 10 IU	246	88.5
	(iii) 10 mg	10	3.6
	(iv) 0.5 IU	8	2.9

Recommended route of oxytocin for management of AMTSL	(i) IV	33	11.9
	(ii) IM	245	88.1

Time of administration of oxytocin for active management of third stage of labor	(i) After delivery of anterior shoulder of baby	6	2.2
	(ii) Within one minute of delivery	246	88.5
	(iii) Within 3 minutes of delivery	14	5
	(iv) More than 3 minutes	12	4.3

How often you perform uterine massage	(i) Every 10 minutes for the 1^st^ 2 hrs	62	22.3
	(ii) Every 15 minutes for the 1^st^ 2 hrs	179	64.4
	(iii) Every 30 minutes for the 1^st^ 2 hrs	31	11.2
	(iv) Every 1 hr for the 1^st^ 2 hrs	6	2.2

**Table 4:** Practice level of midwives towards active management of the third stage of labor in governmental health institutions in the Tigray region, Ethiopia, 2018.

Variables	Response options	Frequency (*n* = 278)	Percentage (%)
Palpate maternal abdomen to rule out presence of another baby	(i) No	98	35.3
	(ii) Yes	180	64.7

Uterotonic drug given for active management of third stage of labor	Oxytocin	278	100

Dose of oxytocin given	10 IU	278	100

Route of oxytocin administration for active management of third stage of labor	IM	278	100

Wait for strong uterine contraction for 2-3 minutes to apply CCT	(i) No	84	30.2
	(ii) Yes	194	69.8

Does not wait for gush of blood to apply CCT	(i) No	93	33.5
	(ii) Yes	185	66.5

Apply CCT correctly	(i) No	79	28.4
	(ii) Yes	199	71.6

Support placenta with both hands during its delivery	(i) No	83	29.9
	(ii) Yes	195	70.1

Extract membrane gently with lateral movement	(i) No	79	28.4
	(ii) Yes	199	71.6

Immediate uterine massage	(i) No	72	25.9
	(ii) Yes	206	74.1

Assess maternal genitalia for tear and trauma	(i) No	71	25.5
	(ii) Yes	207	74.5

Assess completeness of placenta and membrane	(i) No	91	32.7
	(ii) Yes	187	67.3

Ensure uterus does not relax after stopping uterine massage	(i) No	68	24.5
	(ii) Yes	210	75.5

Inform the mother to massage the uterus every 15 minutes for the 1^st^ 2 hrs	(i) No	96	34.5
	(ii) Yes	182	65.5

**Table 5:** Multiple and binary logistic regressions on factors associated with knowledge of midwives towards AMTSL in governmental health institutions in the Tigray region, Ethiopia, 2018.

Variables	Knowledge		Crude OR	Adjusted OR (95% CI)	*p* value
	Good in knowledge	Poor in knowledge			
Sex					
Female	124 (72.9%)	91 (84.3%)	1	1	
Male	46 (27.1%)	17 (15.7%)	1.986	1.518 (0.730, 3.155)	0.264
Workplace					
Health center	47 (27.6%)	50 (46.3%)	1	1	
Hospital	123 (72.4%)	58 (53.7%)	2.256	1.694 (0.841, 3.414)	0.140
Educational status					
Diploma midwives	79 (46.5%)	77 (71.3%)	1	1	
Degree midwives	91 (53.5%)	31 (28.7%)	2.861	1.393 (0.723, 2.682)	0.322
Training					
No	77 (45.3%)	78 (72.2%)	1	1	
Yes	93 (54.7%)	30 (27.8%)	3.140	2.119 (1.141, 3.3937)	0.017^∗^
Years of experience					
0-4	66 (38.8%)	36 (33.3%)	1	1	
5-9	69 (40.6%)	42 (38.9%)	0.636	1.154 (0.540, 2.464)	0.712
Greater than 9 years	35 (20.6%)	30 (27.8%)		0.942 (0.447, 1.983)	0.875
Reading materials					
No	83 (48.8%)	63 (58.3%)	1	1	
Yes	87 (51.2%)	45 (41.7%)	1.467	1.003 (0.511, 1.968)	0.993
Practice level					
Poor in practice	64 (37.6%)	93 (86.1%)	1	1	
Good in practice	106 (62.4%)	15 (13.9%)	10.269	8.089 (4.103, 15.950)	≤0.001^∗^

N.B. 1 = reference category; ^∗^significance at *p* value < 0.05 in multivariate regression.

**Table 6:** Multiple and binary logistic regressions on factors associated with the practice level of midwives towards active management of the third stage of labor in governmental health institutions in the Tigray region, Ethiopia, 2018.

Variables	Practice		Crude OR	Adjusted OR (95% CI)	*p* value
	Good in practice	Poor in practice			
Sex					
Female	88 (72.7%)	127 (80.9%)	1	1	
Male	33 (27.3%)	30 (19.1%)	1.587	1.020 (0.506, 2.058)	0.956
Workplace					
Health center	35 (28.9%)	62 (39.5%)	1	1	
Hospital	86 (71.1%)	95 (60.5%)	1.604	0.689 (0.346, 1.373)	0.290
Educational level					
Diploma midwife	46 (38.0%)	110 (70.1%)	1	1	
Degree midwife	75 (62.0%)	47 (29.9%)	3.816	3.811 (2.015, 7.210)	≤0.001^∗^
Marital status					
Single	24 (19.8%)	45 (28.7%)	1	1	
Married	91 (75.2%)	97 (61.8%)	1.759	1.772 (0.866, 3.626)	0.117
Divorced	4 (3.3%)	14 (8.9%)		0.583 (0.129, 2.639)	0.485
Widowed	2 (1.7%)	1 (0.6%)		2.545 (0.173, 37.422)	0.496
Training					
No	50 (41.3%)	105 (66.9%)	1	1	
Yes	71 (58.7%)	52 (33.1%)	2.867	2.591 (1.424, 4.714)	0.002^∗^
Knowledge level of midwives					
Poor in knowledge	15 (12.4%)	93 (59.2%)	1	1	
Good in knowledge	106 (87.6%)	64 (40.8%)	10.269	7.324 (3.739, 14.393)	≤0.001^∗^

N.B. 2 = reference category; ^∗^significance at *p* value < 0.05 in multivariate regression.

## Data Availability

All data generated/analyzed during this study are included in this published article. Besides, part of the row datasets will be available from the corresponding author on a reasonable request.

## Abbreviations

AOR: Adjusted odds ratio
AMTSL: Active management of the third stage of labor
BEmONC: Basic emergency obstetric and newborn care
CCT: Controlled cord traction
EDHS: Ethiopian Demographic and Health Survey
FIGO: International Federation of Gynecology and Obstetrics
FMOH: Federal Ministry of Health
ICM: International Confederation of Midwives
SPSS: Statistical Package for the Social Sciences
WHO: World Health Organization.

## Additional Points

*Limitations*. Factors like the number of midwives in the delivery room, conduciveness of the delivery room, attitude of midwives towards AMTSL, the health status of study participants, and other unmentioned factors that might affect the knowledge and practice of midwives towards AMTSL were not addressed in this study.
